# Whole Slide Images in Artificial Intelligence Applications in Digital Pathology: Challenges and Pitfalls*

**DOI:** 10.5146/tjpath.2023.01601

**Published:** 2023-05-15

**Authors:** Kayhan Basak, Kutsev Bengisu Ozyoruk, Derya Demir

**Affiliations:** University of Health Sciences, Kartal Dr. Lütfi Kırdar City Hospital, Department of Pathology, Istanbul, Turkey; Bogazici University, Department of Biomedical Engineering, Istanbul, Turkey; Ege University, Faculty of Medicine, Department of Pathology, Izmir, Turkey

**Keywords:** Whole slide images, Artificial intelligence, Digital pathology, Challenges

## Abstract

The use of digitized data in pathology research is rapidly increasing. The whole slide image (WSI) is an indispensable part of the visual examination of slides in digital pathology and artificial intelligence applications; therefore, the acquisition of WSI with the highest quality is essential. Unlike the conventional routine of pathology, the digital conversion of tissue slides and the differences in its use pose difficulties for pathologists. We categorized these challenges into three groups: before, during, and after the WSI acquisition. The problems before WSI acquisition are usually related to the quality of the glass slide and reflect all existing problems in the analytical process in pathology laboratories. WSI acquisition problems are dependent on the device used to produce the final image file. They may be related to the parts of the device that create an optical image or the hardware and software that enable digitization. Post-WSI acquisition issues are related to the final image file itself, which is the final form of this data, or the software and hardware that will use this file. Because of the digital nature of the data, most of the difficulties are related to the capabilities of the hardware or software. Being aware of the challenges and pitfalls of using digital pathology and AI will make pathologists' integration to the new technologies easier in their daily practice or research.

## INTRODUCTION

Artificial intelligence (AI) is a branch of computer science that involves the development of computer algorithms that perform tasks that require human intelligence, such as problem solving, decision-making, visual perception, and pattern recognition (1,2). AI has many potential applications in the field of pathology ranging from pattern analysis in the tissue to detection of patients at the highest cancer risk (3). Overall, AI has the potential to revolutionize the field of pathology by enabling faster and more accurate diagnoses, better treatment decisions, and improved patient outcomes (4–6). The invention of the first commercial slide scanner in 1994 (7) constitutes the turning point for the digital pathology era. With the digitization of the glass slides, analytical calculations can be performed on any image or whole slide images (WSI) by computer-aided systems and subsequently artificial intelligence (AI) algorithms (5,8). The increasing variety of auxiliary diagnostic methods, and the growing number of parameters related to treatment or prognosis make the diagnostic process in the pathology laboratory more and more complex and labor-intensive. AI applications for pathology exceed the limits of imagination, including diagnosis, treatment, prognosis, or histological, and genetic prediction data from any digital data in the form of WSI (3,9–14). Despite the growing number of digital pathology and AI studies in our country, this progress is restricted by the digitization of pathology slides and AI-oriented technical facilities (15). Although a small number of articles (16) with focus on pathology and pathologists' participation are included in national publication directories, many Turkish researchers are involved in international studies and publications (8–10,12,17–19), and the number of publications from Turkey has been increasing (20–25).

The difficulties encountered in AI applications in pathology are diverse and can occur at any stage of the workflow from the pre-analytical phase in the pathology laboratory to AI application (5). At this point, artificial intelligence comes to the scene to simplify the diagnostic pipeline (4). Similar to the microscopic examination of conventional glass slides, the resultant WSIs of AI-supported image analysis applications can be reviewed on computers by pathologists (5). Artifacts can occur at all stages of the routine pathological process and leave traces on the final tissue slide. All these artifacts that interfere with pathological diagnosis are also a problem for AI applications (1,6,12,15,26).

These include all stages of the slide preparation in the pre-analytical and analytical process, conversion of the tissue slides into a digital image file, processing of the digital final image file, and then its use and finalization in the AI application (27). Since the tissue slide is the main source of information to be obtained from pathology, the quality of the slide is the most indispensable fact of diagnosis and the acquisition should be complete and accurate. In the research and development of algorithms, all phases must be carried out with special care and attention (8,26). Finally, most of the difficulties of using the developed AI product for diagnostic support or research can be resolved with end-user training. Being aware of the challenges and pitfalls of digital pathology and artificial intelligence in their daily application or research will facilitate the future daily work of pathologists (9).

## CHALLENGES

The challenges of digital pathology in daily routine can be divided into the pre-acquisition, acquisition, and the post-acquisition period of WSI. Although the challenges are very diverse and encountered at different stages of the process, dividing them into three groups seems appropriate. The difficulties in each phase also depend on the morphological characteristics and the content of the tissue, as well as the technical qualities of the hardware performing the digitization (27,28), the digitization process, and the final image file. During the conversion of glass slides to WSI by scanning, all defects, unwanted tissues, and artifacts that cause optical changes on the glass slide are transferred to the digitized image (28). This is one of the main factors affecting the performance of AI applications. In multicenter studies, additional problems arise from the lack of uniformity in tissue preparation steps. Limitations such as different privacy laws in different countries make multinational studies difficult, even when the method of section preparation method, differences between scanner, patient population, and differences in disease distribution are considered. To eliminate technical problems, models are being developed for different centers to prepare and process their data (29,30).

### Pre-WSI Acquisition Process

The design of the AI application determines the technical sequence and the associated challenges (31,32). Case selection is very important and should consider many subtypes of disease and histologic subtypes, whether neoplastic or non-neoplastic (4). Biopsy samples of the selected disease may be quantitatively or morphologically limited or not sufficient for an AI study. For instance, in the majority of nasopharyngeal carcinoma cases, punch biopsy is performed in which the sampled tissue is small, and almost all further treatment of the patient is planned and carried out based on this biopsy. Nasopharyngeal biopsies usually consist of small and fragmented tissues. This reduces the data that can be used in the AI application and limits the algorithm. Rather, the tissue on the slide may be large, but the target tumor or disease may be very small and/or diffuse. In this case, the annotation that needs to be learned by the AI is tedious and takes a long time, and the data containing the basic information is very small (26). Selected slides contain not only the targeted tumor or lesion, but also include normal tissue, necrosis, cystic spaces, and bleeding areas (32).

Tissue slide quality, collecting slides from slide archives, and selecting the appropriate slides are important prior to the WSI acquisition. After the slide preparation, many external effects create artifacts that may distort the image. Cracks, breaks, and scratches may occur on the slides due to physical exposure. Scratches occur on synthetic coatings during archiving or cleaning and these adversely impact the image quality. Depending on the defects on the slide, melioration may be required such as re-sectioning from the blocks, re-staining, re-covering slides, and cleaning dirty slides. As a result of inappropriate waiting conditions and duration, the slides may become contaminated, and dirty, the applied hematoxylin and eosin (H&E), immunohistochemistry (IHC) and histochemistry (HC) stains may fade, and the cover matrix may dry out. Glass slides with immunofluorescence (IF) and fluorescence in-situ hybridization (FISH) should be digitized without waiting. In addition, these slides are technically much more difficult to convert to WSI final image than H&E slides and require higher technical capability and costly equipment. In research designs on cytological material, there will be no successive imaging of the same cell in smears or liquid-based techniques. In algorithms in which information needs to be obtained with different stains or methods, cytological materials other than the cell block will not be suitable.

### WSI Acquisition

#### Tissue slide dependent challenges

During the scanning of slides for WSI acquisition, artifacts due to many reasons are included as pixels in the final file as misleading information (27). The quality of the tissue slide is indispensable for the quality of the final digital WSI (32). All the problems in the analytical process in the pathology lab affect the quality of the slide, which creates difficulties for the pathologist to reach the final diagnosis. The same losses in tissue slide quality render the final WSI unusable and worthless in AI applications. The problems of the tissue slide may be related to the tissue or may belong to the slide parts other than the tissue. The main problems are that the tissue on the slide is not in the same plane, loss of integrity (scratches), folding, the thickness is too thin or variable, and the stain is darker or pale. Tissue integrity losses are transferred to WSI as they are, causing data and result errors. The focus problem caused by the tissue on the slide not being in the same plane and coverslip thickness can be largely eliminated with the Z-stacking capability of the scanning device (32).

Even if the tissue is ideally processed, sectioned, and stained, there will be difficulties if it is too fragmented, irregularly shaped, too small, or too large. If the tissue is too small or consists of scattered small fragments, annotation and patching will be difficult, laborious, and time-consuming (9,26). Too many slides with wide tissue areas require too much work and too much time and result in too many large files, terabytes of data, in addition to insufficient data transfer rate, storage space, and GPUs. Also, high-resolution images require high-quality and high-processing capacity hardware (4)

#### Device-dependent challenges

**Figure 1:**
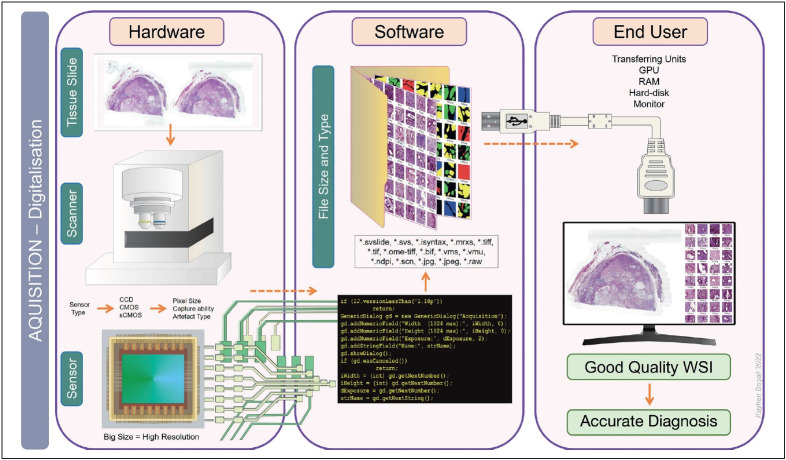
The capacity and capability of the hardware and software determine the type, size, and quality of the final image file during the digitization of the optical image in the tissue slide.

Acquiring high-resolution WSI is essential for accurate diagnosis in digital pathology and AI applications (33). During the acquisition of WSI, there may be problems with the device due to the optical and hardware parts and software that digitize the information from the optical source and sensor. The optical system creates the optical image of the tissue slide. The sensor digitizes the optical data, and the hardware transforms the data into a final image file. The light source and the properties of the transparent parts (lenses, prisms, and mirrors) that make up the optical system are very variable and have a direct impact on the quality of the digitized image. Objective magnification is one of the most important factors affecting the size of the digitized image file. Microns per pixel are directly related to optical magnification and pixel size (27,28). Also, gathering the magnification information for each WSI is a challenge. Even the biggest public dataset TCGA does not include this information. Correct selection and adjustment of the light source will eliminate color defects in the digital image and the need for input normalization before AI application. One of the other essential parts that determine the resolution of the image is the sensor. Sensor characteristics and pixel size not only select image resolution but may result in different types of artifacts during image capture. CCD, CMOS, and sCMOS sensor types show differences in artifact production, imaging ability, and visualization of fluorescent signals. A large sensor provides high resolution. Even if the number, work capacity, and quality of WSI acquisition devices are increasing day by day, high prices constitute problems for the availability of the sources (27) (Figure 1).

Although the high-resolution big-screen viewing monitor looks like the ultimate device in transferring the high-resolution image to the evaluator, high-size image files also require high-capacity memory cards, graphics cards, and larger hard disks. The cost-benefit ratio must be considered regarding hardware, high-speed connection, information technology (IT) infrastructure, and a large amount of data storage costs (long-term storage of glass slides plus whole slide images) (13).

Final image file format, image viewing, transmission, and sharing are dominantly dependent on WSI-acquired scan devices (27). The glass slides can be converted into various digital file types such as .tiff, .jpeg, .tiff, .tiff,. raw, .bif, .vms, .vmu, .ndpi, .scn, .isyntax, .mrxs, .svslide, and .svs based on the scanners (27,32). These files can be visualized by open source WSI viewers such as QuPath, Cytomine, Orbit, ASAP, OpenSlide with OpenSeadragon, ImageJ of SlideJs, PMA.start, and caMicroscope. Open-source solutions are cost-effective for labs and researchers as they allow developers and software engineers to extend and integrate them with their apps. File types are directly related to the viewers that will use them and the file size. This, as expected, is associated with time and hardware overhead in the transmission and sharing of image files (9).

### Post-WSI Acquisition

After getting digitized WSIs, storing them in a structured database in digital pathology laboratories will create an additional financial burden. The workload of storage and digitization will be proportional to the number of patients at the hospital. Providing storage space for digitized data for which the size of one digital slide can range from 1 to 8 GB (34) is another problem, and long-term data protection and maintenance will have an additional cost. The search in the database to select the most suitable and relevant cases will be the next challenge which directly affects the researcher. Determination of the aim of the study and selecting the best fitting histomorphological, histochemical, immunohistochemical, molecular, and oncological data will highly affect the interpretability and impact of the results (26,35).

### Color Standardization and Normalization

Many standardization difficulties in the slide preparation steps in the daily pathology practice cause color variance in the glass slides (36). The variance in the thickness of tissue section, staining materials and methods, illumination conditions, transparent parts, different cells, and extracellular matrix components in different tissues, scanner types, and final viewer devices are accounted for in the main reasons for the difficulty in standardization (4,37). Color differences due to the pre-scan process become more evident, especially in multi-center studies and for long-term archived slides, making color calibration and standardization crucial for robust AI applications (8,32,38). Apart from the color changes and distortions in the glass slides, the colors obtained in the final image WSI may differ from the original slides due to capture parameters such as illumination or any other display factors in the digital systems themselves (37). In terms of the uniformity of terminology, we recommend using calibration terminology to correct device-dependent color distortions during WSI acquisition, standardization to correct color differences in tissue slides, different datasets, and normalization to correct color differences in the same datasets.

Color normalization is essential during the preparation of input and output data in AI applications to get the high-performing models (39). However, AI stain normalization applications (38) face challenges especially for real-time applications: the memory and run-time bottlenecks associated with the processing of images in high resolution, e.g., 40x (33). Moreover, stain normalization can be sensitive to the quality of the input images, e.g., when they contain stain spots or dirt. In this case, the algorithm may fail to accurately estimate the stain vectors and this causes inevitable artifacts during subsequent stitching (40).

Color standardization is one of the most frequent and complex subjects of digital pathology and AI. Color standardization in digital pathology and AI applications cover the final slide, after the scan, and beyond (41). The necessity and method of color standardization should be decided according to the content of the AI application or study (40). The main issues that the authorities should be aware of are: Does the end user see the final image with the corrected color intended and obtained? Does the end user, who sees the final image and decides on the color correction, really see the first and last image colors correctly? To prove this, the user must know the properties of all devices that affect the color from the slide to the final image and have calibrated or corrected them (42,43). This is one of the factors that increase the workload is the necessity of checking the suitability of the color-standardized sections by the pathologists before they are used in the AI application. If the stages of the study are carried out in different institutions or device systems, it is almost impossible to correct the color with a single standard (38). Does AI realize that training and cohort slide groups were different? If it does, is it really because the staining standards in different centers are different?

### Artificial Intelligence Workflow

**Figure 2:**
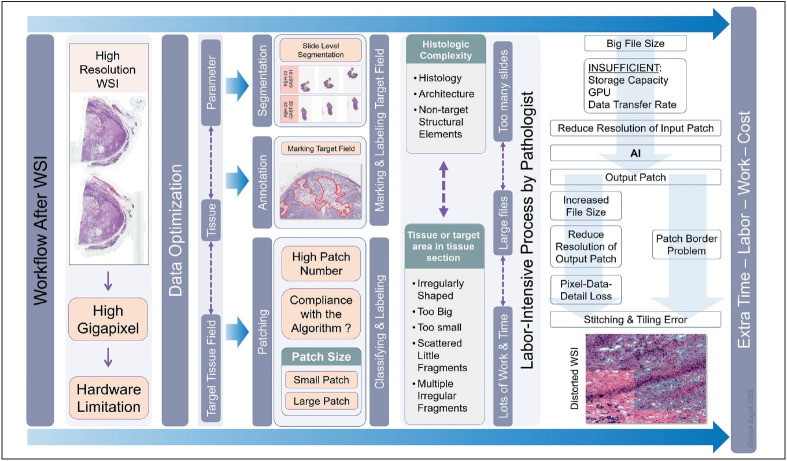
The post-WSI workflow is a complex process that challenges software and hardware abilities. The size and number of images, the capacity, and the capabilities of hardware and software are crucial in AI application efficiency.

The typical WSI processing workflow starts with masking the tissue and non-tissue regions. The size of the whole slide images, reaching up to gigapixels, constitutes the biggest challenge (32). The real time inference and training phase get slower with the increase of the per slide and overall size of the data (32,33). When it is not possible to use WSI due to memory hardware limitations, the images divide into patches and output patches tile. According to the aim of the study, it may not be appropriate to work with patches. As the number of cases, the number of slides, the tissue area in the slide, and the resolution increase, the data to be processed increases proportionally. This increases the number of patches. It becomes impossible for GPUs to process the increasing amount of data. If a GPU with high processing capacity cannot be available, the solution will be to reduce the resolution of the patches. If the parameter you want to find or evaluate is larger than the patch size, or if the size is related or unrelated in more than one patch, this method will result in false negative or positive results. It should be considered in terms of the targeted evaluation of the algorithm (26,44,45). Depending on the algorithm, the concordance between the output patches may be lost at the boundary of the merge (14). This causes a tiling artifact and may appear as patch borders, color differences between patches, and repetitive or disappearing areas in the resulting WSI (Figure 2). Annotation and labeling of the region of interest (ROI) is a frequently used method that reduces the total amount of data by restricting the number of patches and the required GPU capacity. Most of the methods are trained in a supervised manner and require labels in slides or ROI level (17,18). Constructing a huge dataset with annotation is a labor-intensive process that is planned and performed by pathologists (26). The complexity of the structural/histological texture of the tissue or tumor or non-targeted tissue components (erythrocytes, histiocytes) also increases the discordance rate (32). For instance, the simultaneous presence of one or more of the cellular (mitosis, apoptosis, Ki67 proliferation index), textural (invasion, presence of in-situ cancer, different differentiation), or stromal features that are heterogeneously expressed in the tumor tissue will lead to the debate in tumor type determination.

## CONCLUSION and RECOMMENDATIONS

Pathologists, who have been already exposed to an intense daily clinical workload, have little time to devote to research and publish scientific papers on their specialty. Participation in AI studies will bring an additional workload that is not always intolerable for pathologists whose dedicated field of study is not AI. Apart from this, the fear of being replaced by AI applications causes resistance against the integration of decision support tools into daily practice as well as participating in AI research. Adapting pathologists to digital platforms, whose daily practice at hospitals is purely traditional light microscopic evaluation, will require plenty of time and training. Being aware of the technical and morphological difficulties and pitfalls that pathologists may encounter in digital pathology and AI applications will prevent erroneous results and unnecessary waste of time and effort. Whether the availability of AI applications by pathologists provides an advantage to the pathologist or not is dependent on the rate of increase in application efficiency, the associated time, and the context of work performed. In this regard, the perception of difficulties and pitfalls will facilitate the integration of the product and contribute to a high level of utilization of the pathologist.

The most fundamental and indispensable requirement in digital pathology and AI applications is the accuracy and quality of WSI. The challenges for pathologists can be grouped into three: before, during and after the acquisition of the WSI, related to the AI algorithm. Those before acquisition are already in the daily routine of pathology and it is one of the main problems that have been worked on for many years for its standardization. A significant portion of the difficulties in acquiring are device-dependent and can be largely overcome with sufficient budget. Difficulties after WSI may be caused by the device and AI algorithm. Although it is possible to overcome all challenges with device-dependent budgets and algorithm modifications, it is one of the most critical points for algorithm enthusiasts to have full cooperation between the pathologist and the AI software developer and for both to have knowledge of the other’s issues. Finally, ongoing hardware and AI software improvements and more affordable costs will help to overcome challenges more easily.

## Funding

This review did not receive any specific grant from funding agencies in the public, commercial, or not-for-profit sectors.

## Conflict of Interest

Authors have no conflict of interest.
